# Resolution of bleomycin-induced murine pulmonary fibrosis via a splenic lymphocyte subpopulation

**DOI:** 10.1186/s12931-018-0783-2

**Published:** 2018-04-24

**Authors:** Koichiro Kamio, Arata Azuma, Kuniko Matsuda, Jiro Usuki, Minoru Inomata, Akemi Morinaga, Takeru Kashiwada, Nobuhiko Nishijima, Shioto Itakura, Nariaki Kokuho, Kenichiro Atsumi, Hiroki Hayashi, Tomoyoshi Yamaguchi, Kazue Fujita, Yoshinobu Saito, Shinji Abe, Kaoru Kubota, Akihiko Gemma

**Affiliations:** Department of Pulmonary Medicine and Oncology, Graduate School of Medicine, Nippon Medical School, 1-1-5 Sendagi, Bunkyo-ku, Tokyo, 113-8603 Japan

**Keywords:** Regulatory T cells, Idiopathic pulmonary fibrosis, Fibroblast growth factor 9, Interleukin-10, Chemokine (CC motif) ligand-2, Splenectomy

## Abstract

**Background:**

Idiopathic pulmonary fibrosis (IPF) is a progressive disease with high mortality, and the pathogenesis of the disease is still incompletely understood. Although lymphocytes, especially CD4^+^CD25^+^FoxP3^+^ regulatory T cells (Tregs), have been implicated in the development of IPF, contradictory results have been reported regarding the contribution of Tregs to fibrosis both in animals and humans. The aim of this study was to investigate whether a specific T cell subset has therapeutic potential in inhibiting bleomycin (BLM)-induced murine pulmonary fibrosis.

**Methods:**

C57BL/6 mice received BLM (100 mg/kg body weight) with osmotic pumps (day 0), and pulmonary fibrosis was induced. Then, splenocytes or Tregs were adoptively transferred via the tail vein. The lungs were removed and subjected to histological and biochemical examinations to study the effects of these cells on pulmonary fibrosis, and blood samples were collected by cardiac punctures to measure relevant cytokines by enzyme-linked immunosorbent assay. Tregs isolated from an interleukin (IL)-10 knock-out mice were used to assess the effect of this mediator. To determine the roles of the spleen in this model, spleen vessels were carefully cauterized and the spleen was removed either on day 0 or 14 after BLM challenge.

**Results:**

Splenocytes significantly ameliorated BLM-induced pulmonary fibrosis when they were administered on day 14. This effect was abrogated by depleting Tregs with an anti-CD25 monoclonal antibody. Adoptive transfer of Tregs on day 14 after a BLM challenge significantly attenuated pulmonary fibrosis, and this was accompanied by decreased production of fibroblast growth factor (FGF) 9-positive cells bearing the morphology of alveolar epithelial cells. In addition, BLM-induced plasma IL-10 expression reverted to basal levels after adoptive transfer of Tregs. Moreover, BLM-induced fibrocyte chemoattractant chemokine (CC motif) ligand-2 production was significantly ameliorated by Treg adoptive transfer in lung homogenates, accompanied by reduced accumulation of bone-marrow derived fibrocytes. Genetic ablation of IL-10 abrogated the ameliorating effect of Tregs on pulmonary fibrosis. Finally, splenectomy on day 0 after a BLM challenge significantly ameliorated lung fibrosis, whereas splenectomy on day 14 had no effect.

**Conclusions:**

These findings warrant further investigations to develop a cell-based therapy using Tregs for treating IPF.

## Background

Idiopathic pulmonary fibrosis (IPF) is a chronic lung condition that is characterized by progressive scarring of the lung parenchyma [1]. The course of the disease is difficult to predict; however, it generally involves progressive and relentless lung deterioration, curative therapy to quell ongoing fibrosis is not currently available, and the median survival time after diagnosis is 2.5 to 3.5 years [2]. Although the pathological processes underlying disease progression are not fully understood, current paradigms suggest a degenerative process involving alveolar epithelial cell injury and dysregulated repair, leading to aberrant accumulation and proliferation of myofibroblasts, which secrete excessive extracellular matrix (ECM) proteins and ultimately cause fibrotic lung scarring [3, 4]. Although the immunologic factors promoting these events remain unclear, these are important, ongoing areas of research [5, 6].

The significance of lymphocytes in IPF pathogenesis is undetermined. However, lymphocyte infiltration in areas of active fibroblastic proliferation in lungs with IPF have been identified and shown to exhibit an elevated CD4/CD8 T cell ratio that correlates positively with patient survival [3, 7, 8]. Among T lymphocytes, CD4^+^CD25^+^FoxP3^+^ regulatory T cells (Tregs) play pivotal roles in maintaining immune system homeostasis, and significantly impaired Treg activity has been reported among IPF patients [9] although contradictory results have been reported regarding the role of Tregs in lung fibrosis [10, 11]. Supporting Kotsianidis et al. [9], the protective roles of Tregs using antibody-mediated Treg depletion in the fibrotic phase of bleomycin (BLM)-induced murine pulmonary fibrosis were reported [12]. However, whether Tregs ameliorate experimental pulmonary fibrosis in vivo is undetermined and a matter of debate, although BLM-induced murine pulmonary fibrosis cannot completely recapitulate human IPF.

Hence, to better understand the immunological nature of pulmonary fibrosis development, we utilized a well-established, BLM-induced murine pulmonary fibrosis model and investigated the effects of adoptive transfer of splenocytes on fibrosis. In addition, we examined the effect of anti-CD25 antibody-mediated neutralization of CD25 on splenocyte transfer. Moreover, to determine the effects of Tregs during a BLM challenge, we isolated Tregs from spleens and adoptively transferred them into mice with BLM-induced murine pulmonary fibrosis. The effect of the spleen was determined by performing splenectomies at different stages with the current fibrosis model. The current study is the first to describe the effects of adoptive Treg transfer into BLM-induced murine pulmonary fibrosis during the fibrotic phase. Our results will enhance the understanding of Treg-mediated resolution of fibrosis.

## Methods

### Animals

Nine-week-old male C57BL/6 mice were purchased from Charles River Laboratories Japan (Yokohama, Japan). We chose this strain because it is well characterized and is susceptible to BLM-induced pulmonary fibrosis. Interleukin (IL)-10 knock-out mice with C57BL genetic background (B6.129P2-Il10tm1Cgn/J) were purchased from The Jackson Laboratory (Bar Harbor, ME, USA). The experimental protocols in this study were approved by the animal care and use committee of Nippon Medical School (Tokyo, Japan, Approval Number; 27–098).

### BLM treatment and adoptive transfer of splenocytes

**Fig. 1:**
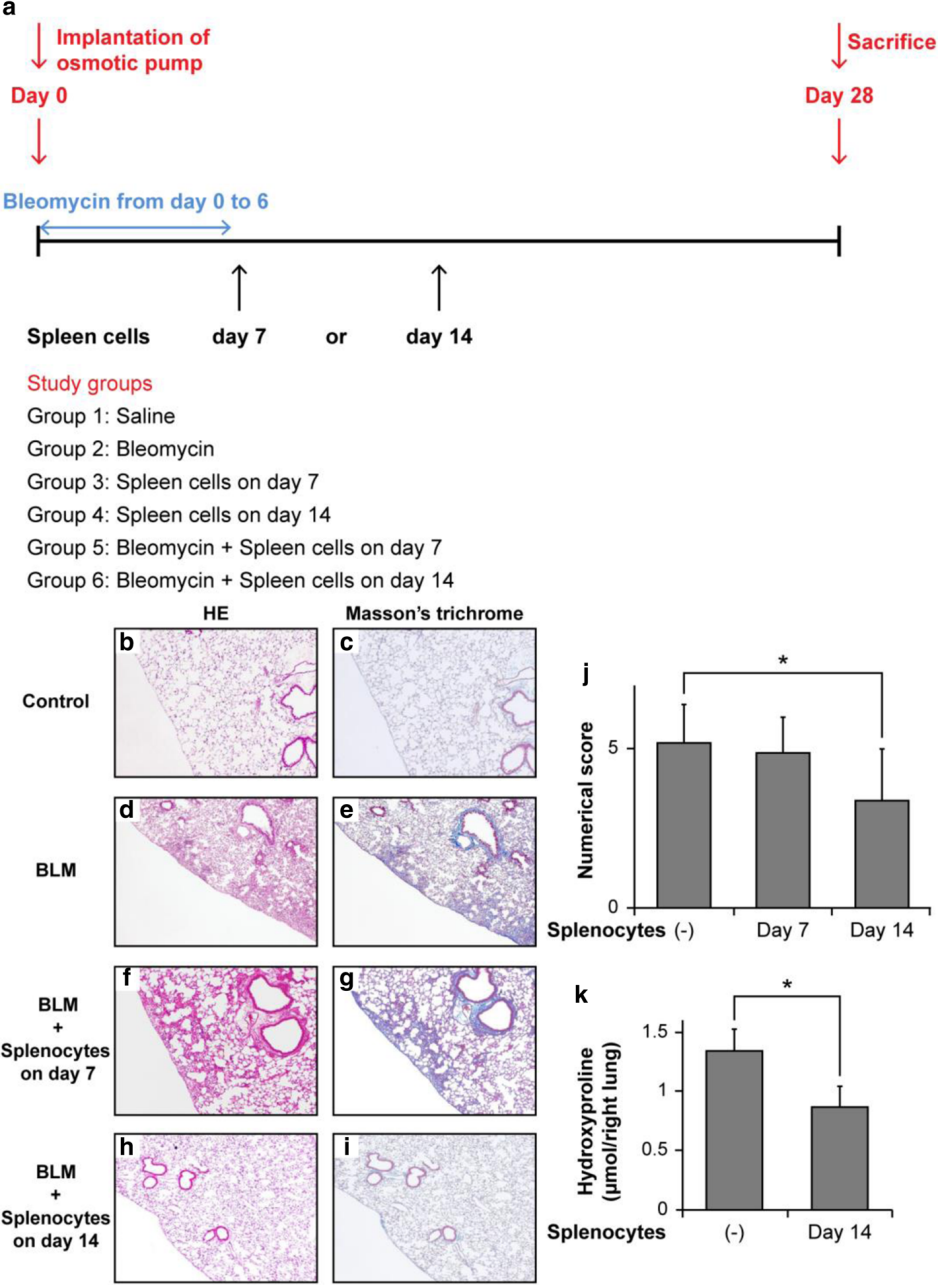
Effect of adoptive transfer of splenocytes on bleomycin (BLM)-induced murine pulmonary fibrosis. **a** Outline of the experimental design used for the adoptive transfer of splenocytes. Osmotic pumps containing 200 μL saline, with or without BLM (100 mg/kg mouse body weight), were implanted subcutaneously through a small incision in the back according to the manufacturer’s instructions. BLM was infused continuously from day 0 to 6. Splenocytes (1 × 10^5^/mouse) were injected via the tail vein either on day 7 or 14 after initiating BLM treatment. On day 28 post-BLM injection, the mice were sacrificed and their lungs were removed for analyses. Each group included at least 6 mice. **b**–**i** Increased fibrosis and collagen deposition observed in the lungs of BLM-treated mice were attenuated by the infusion of splenocytes on day 14 post-BLM treatment. Typical photomicrographs of hematoxylin and eosin (HE) and Masson’s trichrome staining of the left lungs from saline-treated and BLM-treated mice, with or without splenocyte infusion (1 × 10^5^ cells/mouse). Magnification × 10. **j** The extent of lung fibrosis was measured by quantitative histology according to Ashcroft’s method on day 28 to determine the anti-fibrotic effects of splenocytes in the lungs of BLM-treated mice. Because BLM administration with osmotic pumps causes lung fibrosis predominantly in the subpleural regions, subpleural fibrosis between the groups was compared using a numerical scale. Adoptive transfer of splenocytes on day 14 significantly attenuated the numerical score, which was increased by BLM administration. *P < 0.05. *n* = 6–8 mice/group. **k** The hydroxyproline content in the lungs was measured on day 28. Adoptive transfer of splenocytes on day 14 post-treatment significantly reduced the hydroxyproline content in the lungs when compared with the BLM treated group (*P < 0.05). *n* = 3 mice for the BLM group, *n* = 4 mice for the BLM + splenocyte on day 14 group

Osmotic pumps (ALZET model 2001; DURECT Corporation, Cupertino, CA, USA) containing 200 μL saline, with or without BLM (100 mg/kg of mouse body weight; Nippon Kayaku Co., Tokyo, Japan), were implanted in recipient C57BL/6 mice on day 0 (Fig. 1a) [13]. Incision wounds were sealed using a surgical suture. BLM was continuously infused via the pumps over 7 days, according to the manufacturer’s instructions.

Spleens were removed from donor C57BL/6 mice without BLM treatment and minced to obtain single-cell suspensions. Recipient C57BL/6 mice treated with BLM received 1 × 10^5^ donor C57BL/6 splenocytes on day 7 or 14 via caudal vein injection. On day 28, mice were sacrificed and the lungs were removed for examination.

### Histological examination

Lung samples were fixed in 10% formalin buffer (Wako Pure Chemical Industries, Ltd., Osaka, Japan) for histological examination. Paraffin sections (3.5 μm-thick) were cut from fixed lungs, stained with hematoxylin and eosin (HE) to assess gross morphology and Masson’s trichrome stain to visualize collagen deposition [14], and examined by microscopy. Lung fibrosis was measured using quantitative histology following Ashcroft’s method [15].

### Antibody-mediated Treg depletion

To deplete Tregs, we incubated single-cell suspensions of splenocytes (1 × 10^5^) with 1 μL of an anti-CD25 monoclonal antibody (mAb) (clone: PC61; BioLegend, San Diego, CA, USA) dissolved in sterile phosphate-buffered saline (PBS) for 20 min on ice. Then, these cells were adoptively transferred to BLM-treated mice on day 14 via the caudal vein. On day 28, the mice were sacrificed, and the lungs were removed and subjected to HE and Masson’s trichrome staining. The extent of lung fibrosis was measured by quantitative histology according to Ashcroft’s method to determine the effect of CD25 neutralization with the anti-CD25 mAb.

### Fluorescence-activated cell sorting (FACS) analysis of splenocytes

Splenocyte subpopulations were analyzed by evaluating the relative proportions of Tregs, macrophages, and B cells. Single-cell suspensions of splenocytes were stained with anti-mouse CD4 (BioLegend) and CD25 (eBioscience, Waltham, MA, USA) antibodies to analyze Tregs. To analyze macrophages and their polarization, splenocytes were stained with anti-mouse F4/80 (BioLegend), CD80 (eBioscience), and CD206 (BioLegend) antibodies. Splenocytes were stained with an antibody against CD45R (B220; BioLegend) for B cell detection. To eliminate nonspecific staining, isotype- control antibodies, matched to the surface marker antibody’s host species and class, were used. FACS analysis was performed using a BD FACSCanto II and BD FACSVerse flow cytometers (BD Biosciences, San Diego, California, USA). Data collected were analyzed with FlowJo software (Tree Star, Inc., Ashland, Oregon, USA).

### Isolation of Tregs from the spleen and adoptive transfer to BLM-treated mice

After single-cell suspensions were obtained from the spleens of C57BL/6 mice, Tregs were purified using a MiniMacs CD4^+^CD25^+^ Regulatory T-cell Isolation Kit (Miltenyi Biotec, Auburn, CA, USA) according to the manufacturer’s instructions. To achieve the highest purity, we separated positive and negative cell fractions by passing the cells through a second column. BLM-treated recipient C57BL/6 mice received an intravenous tail vein injection of 1 × 10^6^ isolated donor cells on day 14 after initiating BLM treatment. The dose of Tregs was determined according to a study performed by D’Alessio FR and colleagues [16]. Mice were sacrificed on day 28, and the lungs were removed and subjected to histological and biochemical analyses. Blood collected by cardiac punctures and lung homogenates were subjected to cytokine or chemokine analyses.

### Hydroxyproline measurement

The total collagen content of the right lung was determined by hydroxyproline assay [17]. After acid hydrolysis of the right lung with 12 N HCl at 100 °C for 20 h in a sealed glass tube (Iwaki, Tokyo, Japan), the hydroxyproline content was determined by high-performance liquid chromatography.

### Immunohistochemistry for fibroblast growth factor (FGF) 9

Sections of paraffin-embedded lung lobes were deparaffinized and rehydrated. Antigen retrieval was achieved by boiling at 105 °C for 7 min in 10 mM citrate buffer (pH 6.0), followed by gradual cooling to room temperature. Then, the sections were treated with 3% hydrogen peroxide in methanol for 20 min and blocked with 10% normal goat serum (NICHIREI BIOSCIENCES, INC., Tokyo, Japan) at room temperature for 10 min. Sections were incubated with an anti-FGF9 polyclonal antibody (Abcam, Cambridge, MA, USA) for 30 min at room temperature. For FGF9 staining, tissue sections were incubated with a secondary anti-rabbit antibody (NICHIREI BIOSCIENCES, INC.) for 30 min at room temperature. FGF9 expression was visualized using Histofine Simple Stain Mouse MAX-PO (R) and Histofine Simple Stain AEC Solution (NICHIREI BIOSCIENCES, INC.).

### Enzyme-linked immunosorbent assay (ELISA) experiments

Mouse plasma FGF9 and IL-10 levels were measured using ELISA kits purchased from Cloud-Clone Corp. (Houston, TX, USA) and R&D Systems Inc. (Minneapolis, MN, USA), respectively. Approximately 0.5–1.0 mL of blood was collected from each mouse on day 28 by cardiac puncture, placed in a tube containing EDTA and aprotinin, incubated at 4–8 °C for 15 min, and then centrifuged at 5000 rpm for 2 min at 4 °C to separate the plasma. Plasma samples were stored at − 20 °C until analysis. For chemoattractant chemokine (CC motif) ligand 2 (CCL2) measurement in the lungs, the right lung of each mouse was added to 1.0 mL ice-cold lysis buffer (100 mM Tris-HCl pH 7.4, 150 mM NaCl, 1 mM EDTA, and protease inhibitor cocktail [Complete mini; Roche Diagnostics, Basel, Switzerland]), and then homogenates were prepared with a 2-mL tissue grinder (Wheaton Industries, Millville, New Jersey, USA). After centrifuging the homogenate at 10,000×g for 5 min at 4 °C, supernatants were prepared from the lung homogenates, and CCL2 concentration was measured using ELISA kits from Cloud-Clone Corp.

ELISA was performed to determine the concentrations of FGF9, IL-10, and CCL2 following the manufacturer’s protocols. Briefly, supernatants of each sample were diluted in PBS, and 100 μL of the diluted samples was assayed with the kits. The optical density was measured at a wavelength of 450 nm using a microtiter plate reader. All samples were measured in duplicate.

### Immunohistochemistry for col-I and CD45

Sections of paraffin-embedded lung lobes were deparaffinized and rehydrated. Antigen retrieval was achieved by boiling at 105 °C for 7 min in 10 mM citrate buffer (pH 6.0) (LSI Medience Corporation, Tokyo, Japan), followed by gradual cooling to room temperature. Then, the sections were blocked with 1% bovine serum albumin (BSA) (Sigma–Aldrich Corporation, St. Louis, MO, USA) for 20 min at room temperature. Tissue sections were incubated with anti-CD45 and anti-Col-I antibodies (Abcam) overnight at 4 °C followed by Alexa Fluor 488-conjugated and Alexa Fluor 568-conjugated antibodies (Abcam) for 1 h at room temperature. VECTASHIELD mounting medium dispersed over the entire section to counterstain the nuclei with 4′,6-diamidino-2-phenylindole (DAPI) (Vector Laboratories, Inc. Burlingame, CA, USA).

### Immunohistochemistry for FGF9 and E-cadherin

Sections of paraffin-embedded lung lobes were deparaffinized and rehydrated. Antigen retrieval was achieved by boiling at 105 °C for 7 min in 10 mM citrate buffer (pH 6.0), followed by gradual cooling to room temperature. Then, the sections were blocked with 1% BSA for 20 min at room temperature. For FGF9 staining, tissue sections were incubated with an anti-FGF9 antibody (Abcam) for 1 h at room temperature followed by an Alexa Fluor 568-conjugated antibody (Abcam) for 30 min at room temperature. Subsequently, they were blocked with 1% BSA for 20 min at room temperature and incubated with an anti-E-cadherin antibody (Merck KGaA, Darmstadt, Germany) at 4 °C overnight followed by an Alexa Fluor 488 antibody for 30 min at room temperature. VECTASHIELD mounting medium dispersed over the entire section to counterstain the nuclei with DAPI.

### Splenectomy

Either on day 0 or 14 after BLM challenge, the abdominal cavity of mice was opened above the left kidney during isoflurane anesthesia. Then, the spleen vessels were carefully cauterized and the spleen was removed. On day 28, mice were sacrificed and lungs were subjected to HE and Masson’s trichrome staining to quantify the fibrotic score using Ashcroft method.

### Statistical analysis

Animal experiments involved at least 6 mice in each treatment group, unless otherwise stated. Comparisons among multiple groups were analyzed by 1-way analysis of variance with the Tukey–Kramer *post-hoc* correction to adjust for multiple comparisons. An unpaired 2-tailed Student’s *t* test was used for single comparisons. Data were analyzed using JMP 9 software, version 9.0.3 (SAS Institute Inc., Cary, NC, USA). Differences were considered statistically significant when *P* values were less than 0.05. Data are expressed as the mean ± standard deviation.

## Results

### Resolution of BLM-induced murine pulmonary fibrosis by the adoptive transfer of splenocytes

Because whole splenocytes can ameliorate LPS-induced lung injury in mice [16], as an initial assessment, we examined the potency of the adoptive transfer of splenocytes in ameliorating BLM-induced murine pulmonary fibrosis. BLM (100 mg/kg body weight) was administered to C57BL/6 mice using osmotic pumps (day 0, Fig. 1a). Splenocytes were prepared from the spleens of C57BL/6 mice without BLM treatment and injected (1 × 10^5^ cells/mouse) via the caudal vein on either day 7 or 14 post-BLM challenge. Mice were sacrificed on day 28, and the lungs were removed and subjected to histological examination. Lung histological data obtained on day 28 post-BLM administration showed focal fibroplasias with destruction of the alveolar wall in the group receiving BLM (Fig. 1d and e) but not in the saline group (Fig. 1b and c). Injection of splenocytes on day 14 ameliorated the lesions (Fig. 1h and i); however, no obvious effect of splenocyte injection was observed on day 7 (Fig. 1f and g). To quantify the anti-fibrotic effects of splenocytes in the lungs of BLM-treated mice, we determined the extent of lung fibrosis by quantitative histology according to Ashcroft’s method on day 28 post-treatment. Because BLM administration with osmotic pumps causes lung fibrosis predominantly in the subpleural regions [18, 19], subpleural fibrosis between the groups was compared using a numerical scale. Two blinded observers [KKa and MI] quantified fibrosis in each section. Splenocytes significantly attenuated the fibrosis score when administered to BLM-treated mice on day 14 (Fig. 1j, *P < 0.05); however, administration of splenocytes on day 7 had no effect on pulmonary fibrosis. To characterize the anti-fibrotic effects of splenocytes, we assayed the hydroxyproline content in the lungs on day 28. As shown in Fig. 1k, the adoptive transfer of splenocytes on day 14 post-BLM challenge significantly reduced the hydroxyproline content in the lungs when compared with the BLM treated group (*P < 0.05).

### Effect of splenocytes depleted of Tregs on BLM-induced murine pulmonary fibrosis

**Fig. 2:**
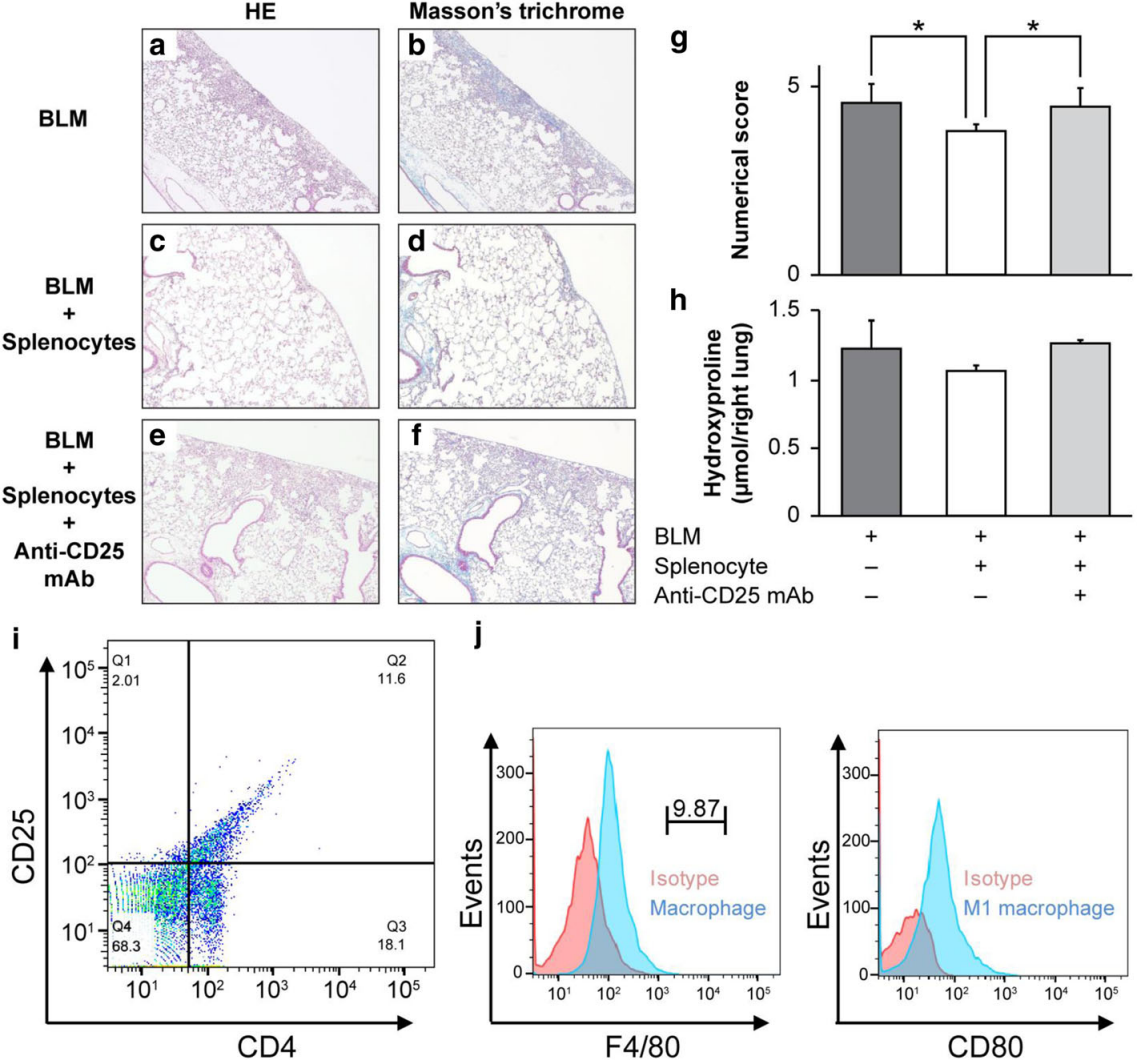
Effect of splenocytes depleted of Tregs on bleomycin (BLM)-induced murine pulmonary fibrosis. **a**–**f** Splenocyte suspensions (1 × 10^5^ cells) obtained from C57BL/6 mice were incubated with 1 μL of an anti-CD25 monoclonal antibody (mAb) for 20 min on ice, after which they were injected via the caudal vein on day 14 post-BLM treatment. On day 28, the mice were sacrificed and the lungs were removed and subjected to hematoxylin and eosin (HE) and Masson’s trichrome staining. **g** The extent of lung fibrosis was measured by quantitative histology according to Ashcroft’s method to determine the effect of CD25 neutralization with the anti-CD25 mAb. Treatment of splenocytes with the anti-CD25 mAb abrogated the anti-fibrotic effect of splenocytes in BLM-induced pulmonary fibrosis. *P < 0.01. *n* = 7 mice/group. **h** The hydroxyproline content in the lungs was measured on day 28. A similar trend in the numerical score was observed in the lung hydroxyproline content, although statistical significance was not achieved. n = 4 mice for each group. Splenocytes were subjected to FACS analysis to identify Tregs (**i**) and macrophages (**j**). Tregs accounted for approximately 11% of the splenocytes. Macrophages consisted of approximately 10% splenocytes, most of which had the M1 phenotype

Splenocytes have been reported to be rich in Tregs, and D’Alessio et al. reported that Tregs contributed to the resolution of LPS-induced lung injury in mice [16]. Given that the resolution of BLM-induced murine pulmonary fibrosis was dependent on spleen cells due to their suppressive properties, we hypothesized that specific lymphocyte subsets among splenocytes may be critical in this process. To begin understanding the potential contribution of those cells to BLM responses, we depleted Tregs using an anti-CD25 mAb. Splenocytes were incubated with the anti-CD25 mAb, after which they were injected via the caudal vein on day 14 post-BLM treatment. On day 28, the mice were sacrificed, and their lungs were removed and subjected to histological examination and biochemical analyses (Fig. 2). As shown in Fig. 2e and f, splenocytes depleted of Tregs using an anti-CD25 mAb lost the potential to ameliorate BLM-induced murine pulmonary fibrosis. To quantify this effect, we determined the extent of lung fibrosis by quantitative histology on day 28, according to Ashcroft’s method. Two blinded observers [KKa and MI] quantified the degree of fibrosis in each section. Amelioration of BLM-induced murine pulmonary fibrosis by splenocytes was significantly abrogated by Treg depletion with the anti-CD25 mAb (Fig. 2g, * *P* < 0.01). These data indicated that CD25-mediated Treg depletion promotes increased lung fibrosis, thereby suggesting that immunomodulatory cells (including Tregs) might mediate the effects of splenocytes on pulmonary fibrosis resolution. A similar trend in the numerical score was observed on the lung hydroxyproline content, although statistical significance was not achieved (Fig. 2h, *P* = 0.16).

To characterize splenocytes, we examined the number of CD4 and CD25 double-positive cells by immunostaining and FACS analysis of spleen digests from BLM-untreated C57BL/6 mice. As shown in Fig. 2i, Tregs accounted for approximately 11% of the splenocytes. Furthermore, we assayed macrophage subpopulations because diverse activation states of macrophages can result in phenotypic polarization and the formation of pro-inflammatory M1 and pro-healing M2 type macrophages [20]. As shown in Fig. 2j, approximately 10% of splenocytes were macrophages (F4/80), most of which were of the M1 phenotype, in agreement with previous data published elsewhere [21]. B cells constituted approximately 30% of the splenocytes (data not shown).

### Adoptive transfer of isolated Tregs to BLM-treated mice with pulmonary fibrosis

**Fig. 3:**
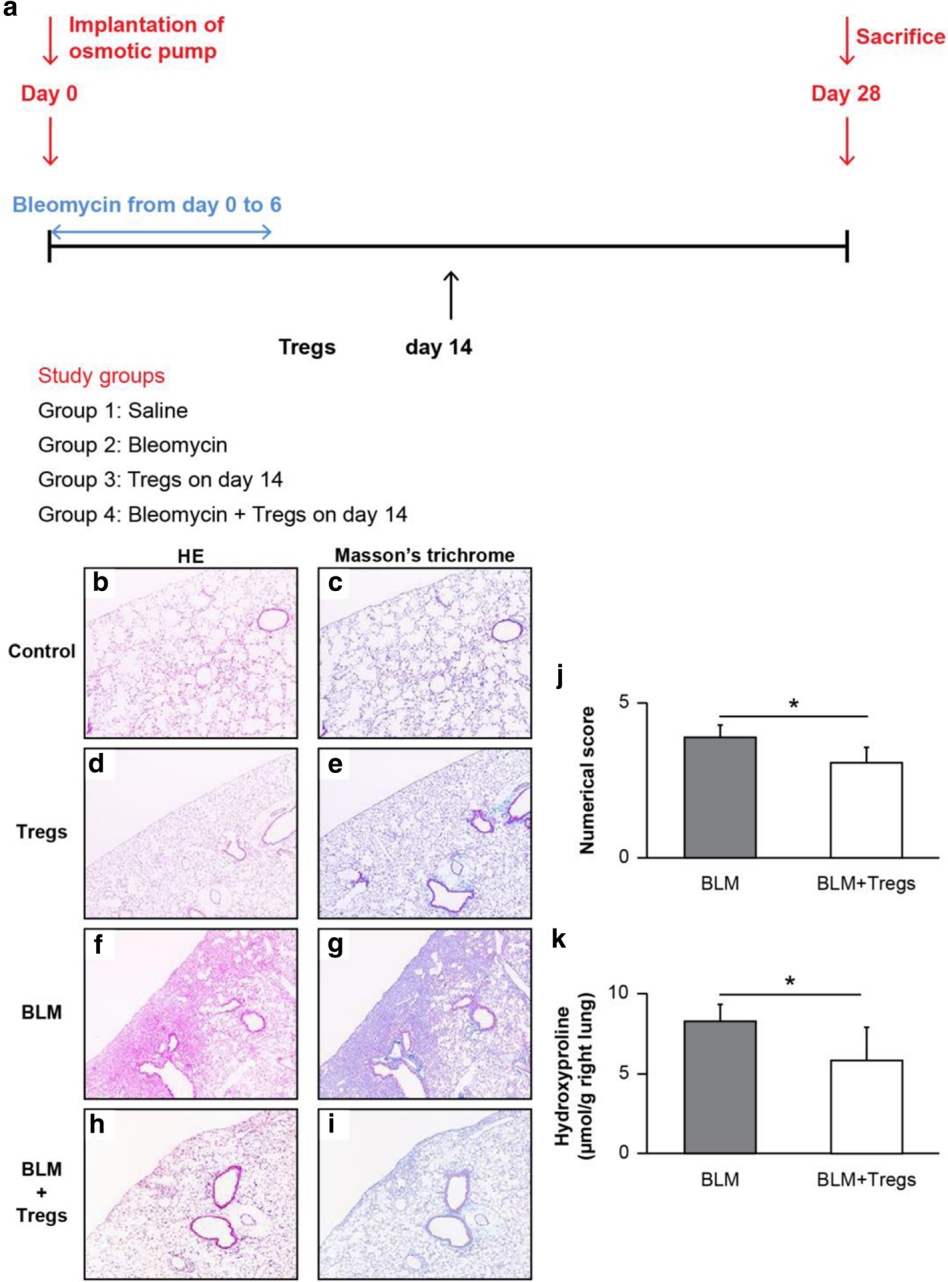
Effect of adoptively transferring Tregs on bleomycin (BLM)-induced murine pulmonary fibrosis. **a** Outline of the experimental design used for the adoptive transfer of Tregs. Osmotic pumps containing 200 μL saline, with or without bleomycin (BLM; 100 mg/kg mouse body weight), were implanted subcutaneously through a small incision in the back according to the manufacturer’s instructions. BLM was infused continuously from day 0 to 6. Tregs (1 × 10^6^/mouse) were injected via the tail vein on day 14 after initiating BLM treatment. On day 28 post-BLM challenge, the mice were sacrificed and their lungs were removed and blood was collected for analyses. Each group included at least 6 mice unless otherwise stated. **b**–**i** Increased fibrosis and collagen deposition observed in the lungs of BLM-treated mice were attenuated by the adoptive transfer of Tregs (1 × 10^6^/mouse) on day 14 post-BLM challenge. Representative photomicrographs following hematoxylin and eosin (HE) and Masson’s trichrome staining of the right lungs from saline-treated and BLM-treated mice, with or without adoptively transferred Tregs. Magnification × 40. **j** The extent of lung fibrosis was measured by quantitative histology according to Ashcroft’s method on day 28 to determine the anti-fibrotic effects of Tregs in the lungs of BLM-treated mice. The adoptive transfer of Tregs on day 14 post-treatment significantly attenuated the numerical score, which was increased by BLM treatment. *P < 0.01. n = 7 mice/group. **k** The hydroxyproline content in the lungs was measured on day 28. The adoptive transfer of Tregs on day 14 post-treatment significantly reduced the hydroxyproline content when compared with the BLM treated group. *P < 0.05. *n* = 4 mice for the BLM group, *n* = 3 mice for the BLM + Tregs group

The data obtained using the anti-CD25 mAb prompted us to examine whether Tregs potently resolve BLM-induced pulmonary fibrosis in mice. To that end, Tregs isolated from spleens of donor C57BL/6 mice were adoptively transferred to recipient mice (1 × 10^6^ cells/mouse) via the caudal vein on day 14 post-BLM challenge (Fig. 3a). Because adoptive transfer of splenocytes on day 14 ameliorated fibrosis, which was abrogated by an anti-CD25 antibody, this time point was selected for the Treg transfers. Thereafter, on day 28, mice were sacrificed, and the lungs were removed and subjected to immunohistochemical and biochemical analyses (Fig. 3). In addition, blood was collected by cardiac punctures to analyze the FGF9 and IL-10 levels, and lung homogenates were prepared to measure CCL2 levels. As shown in Fig. 3h and i, adoptively transferring Tregs on day 14 ameliorated the lesions. To quantify the anti-fibrotic effects of Tregs in the lungs of BLM-treated mice, we determined the extent of lung fibrosis using a quantitative histology according to Ashcroft’s method on day 28. Two blinded observers [KKa and AM] quantified fibrosis in each section. Tregs significantly attenuated the fibrosis score when adoptively transferred to BLM-treated mice on day 14 (Fig. 3j, *P < 0.01). To further characterize the anti-fibrotic effects of Tregs, we quantified the hydroxyproline content in the lungs on day 28. As shown in Fig. 3k, adoptive transfer of Tregs significantly reduced the hydroxyproline content in the lungs when compared with the BLM treated group (*P < 0.05).

### Effect of adoptively transferred Tregs on FGF9 expression in BLM-induced murine pulmonary fibrosis

**Fig. 4:**
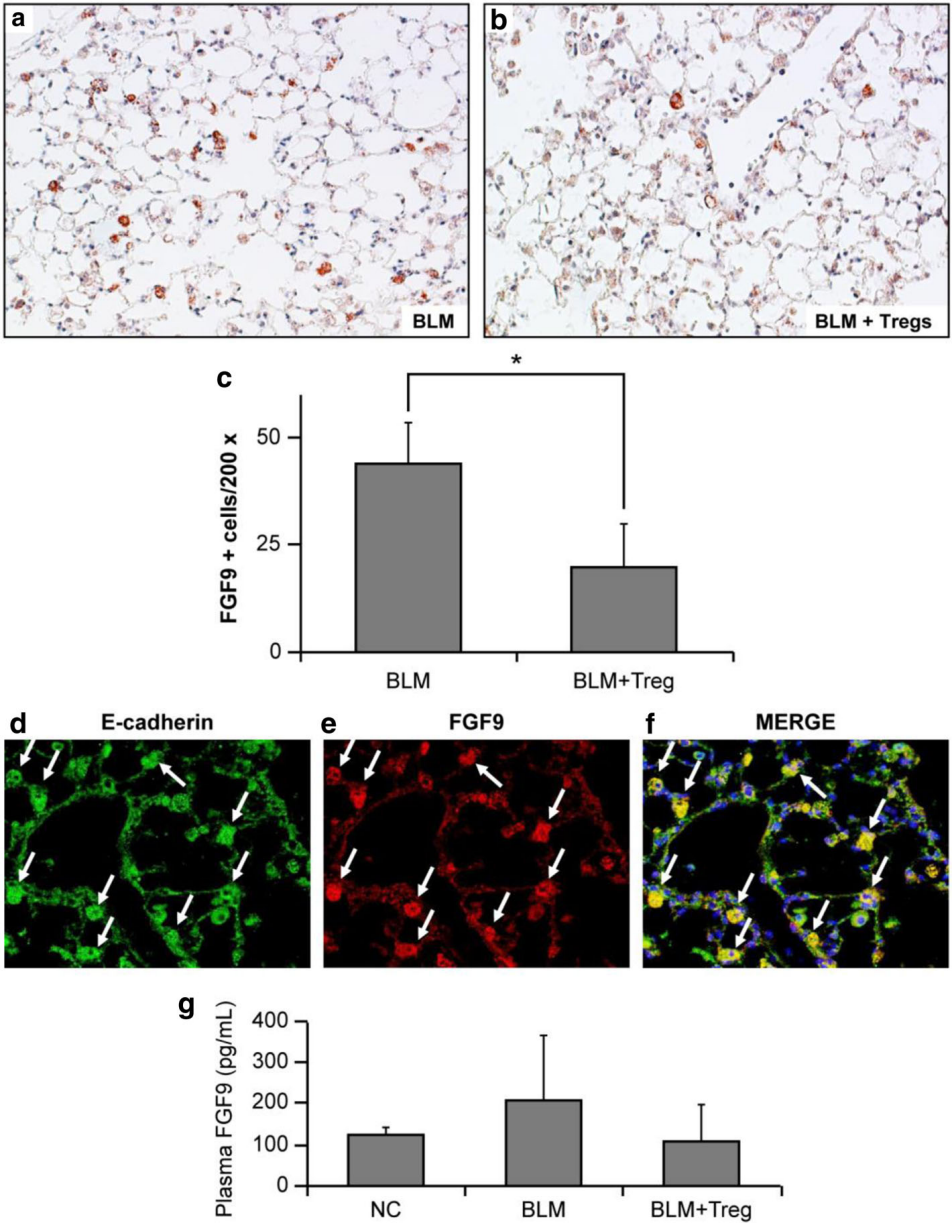
Adoptively transferred Tregs ameliorated the accumulation of fibroblast growth factor (FGF) 9-expressing cells. Immunohistochemical staining for FGF9 (red) in mouse lungs with BLM-induced pulmonary fibrosis, without or with adoptively transferred Tregs (**a** and **b**, respectively). FGF9-positive cells bearing the morphology of alveolar epithelial cells markedly increased by BLM treatment (**a**), and this increase was attenuated by the adoptive transfer of Tregs (**b**). Tissue sections were counterstained with hematoxylin. Representative data from each group are presented. **c** Composite data represented as the number of FGF9-positive cells per 200× field. n = 3 for BLM, n = 4 for BLM + Tregs. *P < 0.05. **d**–**f** Dual staining for E-cadherin (green; **d**) and FGF9 (red; **e**) was performed with lung sections on day 28 after BLM treatment. The merged images (yellow; **f**) represent co-staining for E-cadherin and FGF9, indicating that both, FGF9 and E-cadherin, are expressed by alveolar epithelial cells. **g** Plasma samples from BLM-treated and Treg-transferred mice were collected by cardiac punctures on day 28 post-BLM challenge, and FGF9 levels were measured by ELISA. The plasma FGF9 concentration appeared to increase following BLM treatment. A trend was observed that the increase tended to be reduced to basal levels by the adoptive transfer of Tregs. n = 3–4 mice/group. NC; normal control

FGF9 is a neuroimmune system molecule with manifold effects on embryonic development and mesenchymal proliferation that may be involved in human lung fibrosis [22]. While in vivo data demonstrated that antibody-mediated Treg depletion augmented FGF9 expression in mice overexpressing TGF-β1 [23], the effect of adoptively transferred Tregs on FGF9 expression in BLM-induced lung fibrosis is not yet described. To determine whether Tregs affect FGF9 expression, we performed immunohistochemistry against FGF9. The control and Treg transferred groups had undetectable FGF9 expression (data not shown), which was consistent with a prior report [23]. In contrast, FGF9-positive cells bearing the morphology of alveolar epithelial cells markedly increased in the setting of BLM-induced pulmonary fibrosis (Fig. 4a), whereas FGF9-positive cells decreased following the adoptive transfer of Tregs (Fig. 4b). Composite data represented as the number of FGF9-positive cells revealed that the adoptive transfer of Tregs reduced FGF9-positive cells by 54.2%, as compared to BLM-treated mice without adoptively transferred Tregs (Fig. 4c). To confirm that FGF9-positive cells are epithelial cells, we performed dual staining of these cells using anti-FGF9 and anti-E-cadherin antibodies. As shown in Fig. 4d–f, most of the FGF9-positive cells (red) expressed E-cadherin (green). In addition, we determined the plasma FGF9 levels by ELISA. As shown in Fig. 4g, the plasma FGF9 concentration appeared to increase with BLM treatment, as compared to saline-treated control mice. The increase showed a trend to be reduced to control levels by the adoptive transfer of Tregs, although statistical significance was not achieved.

### Effect of Tregs on a soluble mediator of inflammation

**Fig. 5:**
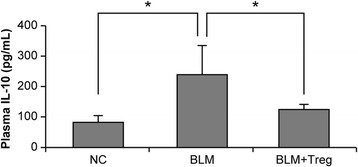
Determination of interleukin (IL)-10 plasma concentrations in mice. Plasma samples from bleomycin (BLM)-treated and Treg-transferred mice were collected by cardiac punctures on day 28 after initiating BLM administration, after which their IL-10 levels were measured by ELISA. Plasma IL-10 concentrations significantly increased by up to 3.0-fold after BLM-treatment. This increase was reversed to basal levels by the adoptive transfer of Tregs. *P < 0.05. *n* = 5 mice/group. NC; normal control

Tregs are conventionally associated with the production of classic anti-inflammatory cytokines including IL-10, TGF-β, and IL-35, consistent with their anti-inflammatory functions [24]. Among these cytokines, IL-10 has been reported to inhibit BLM-induced lung injury in mice [25, 26]. Therefore, we hypothesized that the Treg-mediated anti-fibrotic effect was due in part to IL-10 production from adoptively transferred Tregs. To test this hypothesis, we collected plasma samples from BLM-treated and Treg-transferred mice, and then assessed IL-10 levels in ELISAs. The data indicated that the plasma IL-10 concentration increased by up to 3.0-fold after BLM treatment (Fig. 5). Unexpectedly, the adoptive transfer of Tregs significantly inhibited IL-10 production in mouse plasma induced by BLM administration.

### Effect of Tregs from IL-10 knock-out mice on BLM-induced murine pulmonary fibrosis

**Fig. 6:**
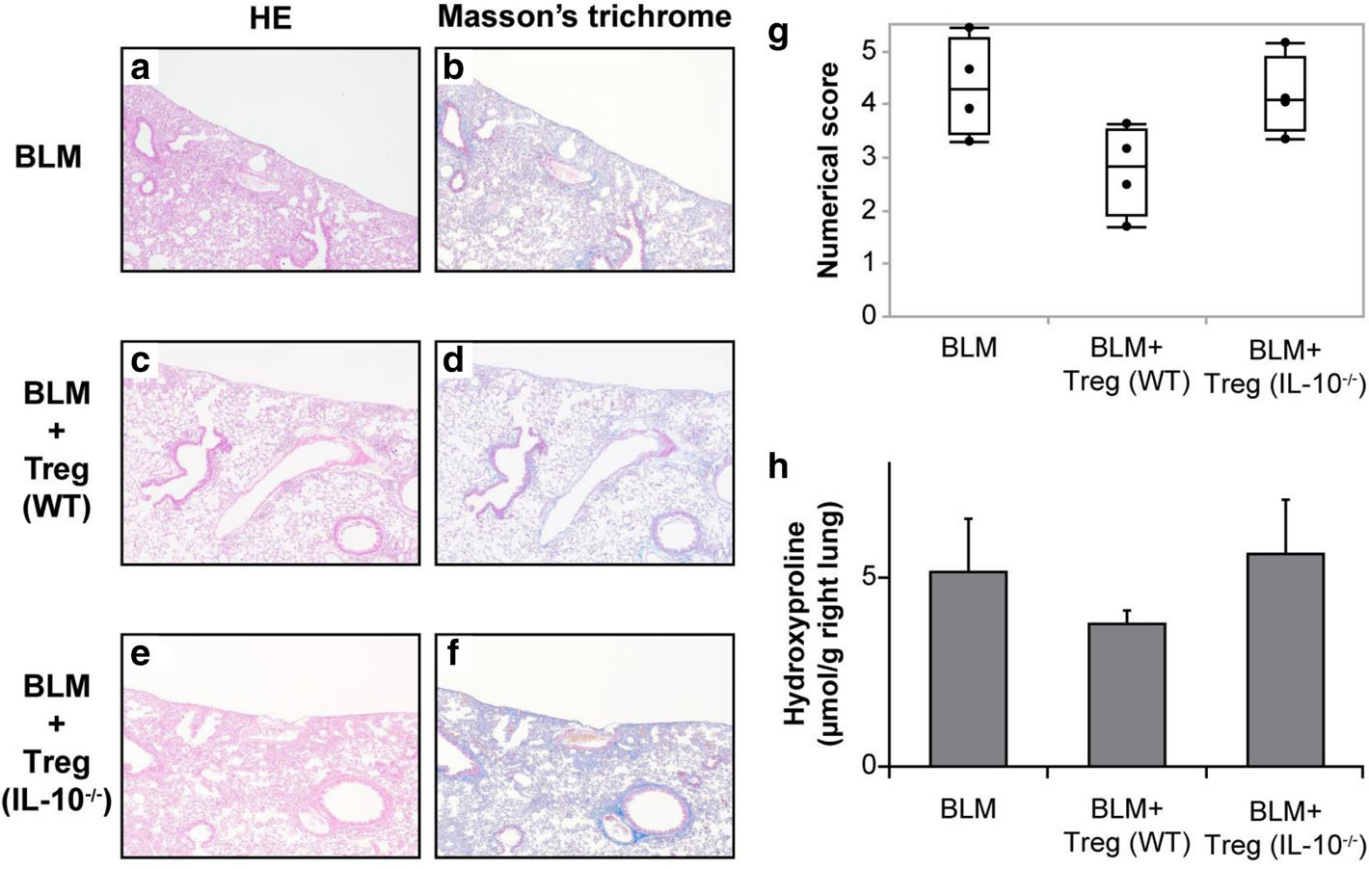
Effect of adoptively transferring Tregs isolated from interleukin (IL)-10 knock-out mice on pulmonary fibrosis. Tregs isolated from wild-type and IL-10 knock-out mice were injected via the tail vein (1 × 10^6^/mouse) on day 14 after initiating BLM treatment. On day 28 post-BLM challenge, the mice were sacrificed and their lungs were removed for analyses. **a**–**f** Adoptive transfer of Tregs isolated from IL-10 knock-out mice did not attenuate fibrosis and collagen deposition induced by BLM treatment. Representative photomicrographs following hematoxylin and eosin (HE) and Masson’s trichrome staining are presented. Magnification × 40. **g** The extent of lung fibrosis was measured on day 28 by quantitative histology following Ashcroft’s method to determine the anti-fibrotic effects of Tregs obtained from both wild-type and IL-10 knock-out mice. Adoptive transfer of Tregs isolated from IL-10 knock-out mice did not ameliorate the lesion. Data are displayed as median and interquartile range. **h** The hydroxyproline content in the lungs was measured on day 28. A similar trend for the numerical score was observed. n = 4 mice/group. WT; wild-type, IL-10−/−; IL-10 knock-out

The above data prompted us to examine the effect of Tregs isolated from IL-10 knock-out mice on pulmonary fibrosis. Tregs isolated from IL-10 knock-out mice spleens were adoptively transferred via the tail vein on day 14 after a BLM-challenge according to the protocol described in Fig. 3a. On day 28, the mice were sacrificed, and the lungs were removed and subjected to immunohistochemical and biochemical analyses (Fig. 6). As shown in Fig. 6e and f, adoptive transfer of Tregs isolated from IL-10 knock-out mice did not ameliorate the fibrosis. We quantified these results using quantitative histology according to Ashcroft’s method on day 28. Two blinded observers [KKa and AM] quantified fibrosis in each section. Although statistical significance was not achieved, a trend was observed indicating that the transfer of Tregs isolated from IL-10 knock-out mice did not attenuate the fibrosis score when compared with transferring Tregs obtained from wild-type C57BL/6 mice (Fig. 6g). We also quantified the hydroxyproline content in the lungs on day 28. As shown in Fig. 6h, a similar trend for the numerical score was observed.

### Effect of Tregs on fibrocyte chemoattractant CCL2 production and fibrocyte accumulation in the lungs

**Fig. 7:**
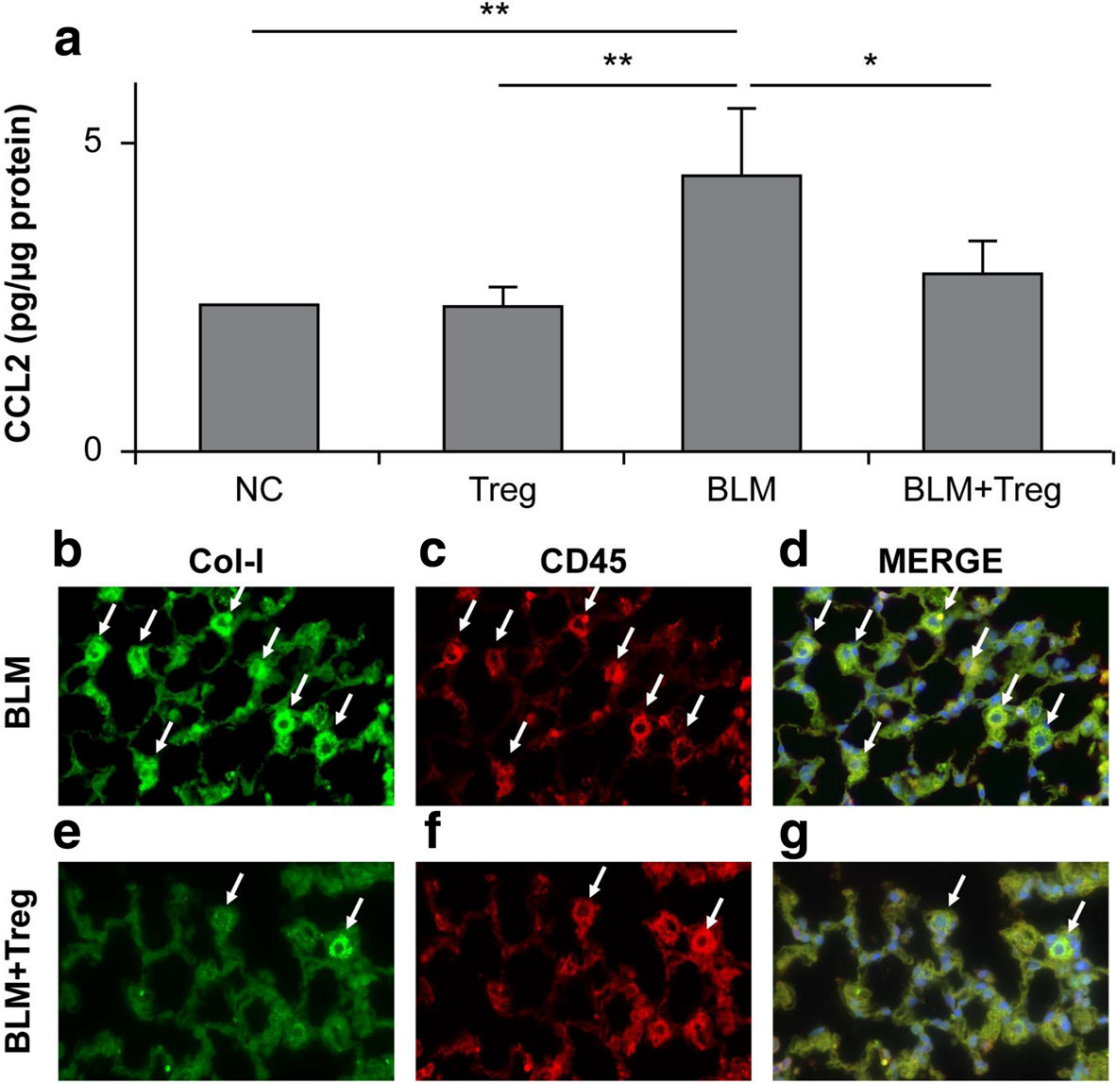
Adoptively transferred Tregs reduced chemokine (CC motif) ligand-2 (CCL2) production and fibrocyte accumulation. **a** CCL2 levels in lung digests were measured by performing ELISA on day 28 post-BLM challenge. CCL2 production was significantly increased in BLM-treated mouse lungs compared with that in lungs from saline-treated and Treg-transferred mice. Adoptive transfer of Tregs significantly attenuated CCL2 production in BLM-treated mice. *P < 0.05, ** *P* < 0.01. n = 3 mice/group. NC; normal control. **b**–**g** Immunohistological analyses for collagen-I (Col-I; green; **b** and **e**) and CD45 (red; **c** and **f**) were performed with lung sections on day 28 after BLM treatment, with or without adoptive Treg transfer. The merged images (yellow; **d** and **g**) represent co-staining for Col-I and CD45, indicating the presence of bone marrow-derived fibrocytes. Fibrocytes are indicated with arrows. The increased numbers of fibrocytes expressing Col-I and CD45 observed in BLM-treated mice were attenuated by adoptive Treg transfer. Nuclei were counterstained with 4′,6-diamidino-2-phenylindole. Original magnification, × 40

Bone marrow-derived circulating fibrocytes are mesenchymal progenitor cells that express markers compatible with leukocytes, hematopoietic progenitor cells, and fibroblasts [27]. They are chemotactically recruited to sites of tissue injury by chemokines, including CCL2 [28], and promote fibrotic responses [29]. Because Tregs have been found to inhibit fibrocyte recruitment [23], we hypothesized that Treg adoptive transfer affects chemokine production in the lungs. To determine this, we measured CCL2 production by ELISA in BLM-induced murine pulmonary fibrosis lungs that had received Treg adoptive transfer. As shown in Fig. 7a, BLM treatment increased CCL2 production by up to 2.0-fold in the lungs. Adoptive transfer of Tregs significantly reversed this increase to the basal levels, which was accompanied by reduced accumulation of fibrocytes in the lungs (Fig. 7b–g).

### Effect of splenectomy on BLM-induced murine pulmonary fibrosis

**Fig. 8:**
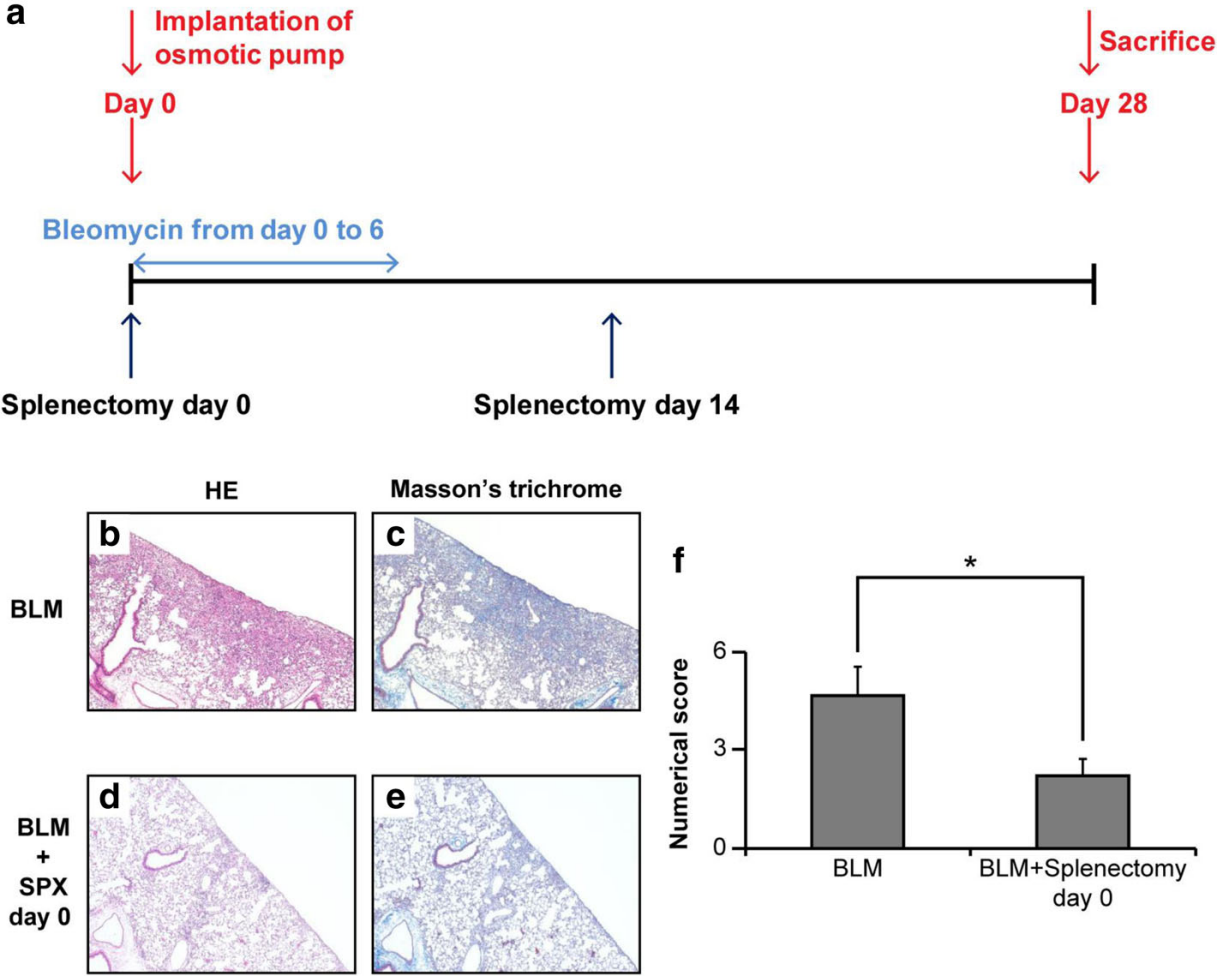
Effect of splenectomies on bleomycin (BLM)-induced murine pulmonary fibrosis. Outline of experimental design used for splenectomies **a**. Osmotic pumps containing 200 μL saline, with or without BLM were implanted subcutaneously through a small incision in the back according to the manufacturer’s instructions. Then, spleen vessels were cauterized and the spleen was removed either on day 0 or 14. On day 28, mice were sacrificed and the right lungs were subjected to hematoxylin and eosin (HE) and Masson’s trichrome staining. The extent of lung fibrosis was measured by quantitative histology, according to Ashcroft’s method. Splenectomy on day 0 significantly ameliorated pulmonary fibrosis (**b**–**f**). Mice that underwent a splenectomy on day 14 were administered 60 mg BLM/kg body weight to reduce the mortality caused by splenectomy on day 14. *P < 0.05. n = 5 mice/group. SPX; splenectomy

Mouse splenocytes can be reduced in number by BLM administration [30], causing us to speculate that spleen is a reservoir of Tregs. Because our observation suggested the participation of spleen cells in the amelioration of pulmonary fibrosis, we were prompted to examine the effect of splenectomy. BLM was administered to C57BL/6 mice using osmotic pumps (day 0, Fig. 8a), after which the spleen vessels were carefully cauterized and spleen was removed either on day 0 or 14. On day 28, the mice were sacrificed and the lungs were subjected to HE and Masson’s trichrome staining to quantify the fibrotic score, using Ashcroft’s method. Two blinded observers [KKa and AM] quantified the degree of fibrosis in each section. As shown in Fig. 8b–f, splenectomy on day 0 significantly ameliorated pulmonary fibrosis, whereas that on day 14 had no effect on fibrosis (data not shown).

## Discussion

Here, we demonstrated for the first time (to our knowledge) that splenocytes can potently ameliorate pulmonary fibrosis in vivo, using a well-characterized, BLM-induced murine pulmonary fibrosis model. This effect was abolished by antibody-mediated Treg depletion. We further observed that BLM-induced lung fibrosis was attenuated by adoptive Treg transfer when they were transferred at the fibrotic stage, using our model. This effect was accompanied by Treg-mediated suppression of FGF9 and CCL2 production. Moreover, splenectomy performed simultaneously with BLM administration ameliorated fibrosis formation. These findings may provide evidence of an integral role for Tregs in the resolution of pulmonary fibrosis, which we believe to be a novel finding. These findings are expected to broaden our understanding of immunological mechanisms underlying the disease.

The role of inflammation along with the involvement of the immune response remains largely obscure in IPF pathogenesis. The implication of T cells in IPF development is controversial. Instead, evidence suggests that T lymphocytes may modulate inflammatory and healing responses in the lungs in a profibrotic or antifibrotic manner depending on their phenotype, and the balance between these functionally different T cell subsets may be a determining factor in the development of fibrosis [10]. Immunosuppressive Tregs represent a novel T cell sub-population that is expected to act profibrotically [10]. Kotsianidis and colleagues, however, reported that in patients with IPF, Tregs were reduced compared with those of healthy volunteers and patients without IPF, and Treg activity was significantly impaired [9]. In support of these observations, Shimizu and colleagues showed that interstitial Foxp3-positive lymphocytes decreased in the lungs of patients with usual interstitial pneumonia [31]. Antithetically, Galati and colleagues found that Tregs were significantly increased in patients with IPF [11]. Thus, the roles that Tregs play in IPF development are still under debate.

These issues are similarly controversial in lung fibrosis animal models, and conflicting results have been found. In this study, Tregs adoptively transferred on day 14 post-BLM treatment significantly ameliorated BLM-induced murine pulmonary fibrosis; however, the role of Tregs on pulmonary fibrosis is still a matter of debate, as exemplified by a study in which Treg depletion attenuated the development of silica-induced lung fibrosis in mice [32]. The discrete triggers involved in inducing lung fibrosis may partly explain these disparities. Consistent with our observations, Boveda-Ruiz and colleagues demonstrated that Treg depletion accelerated pulmonary fibrosis during the late stage of BLM-induced murine pulmonary fibrosis [12]. However, they also demonstrated that Treg depletion during the early stage in their model ameliorated pulmonary fibrosis, similar to results from Birjandi et al. where the adoptive transfer of Tregs aggravated BLM-induced lung fibrosis in mice when administered during early inflammatory phase [33]. In general, BLM administration induces acute pan-alveolitis during the first week, and the inflammatory response peaks on day 7 after beginning drug infusion [34, 35]. This early phase is followed by a late process characterized by enhanced myofibroblast proliferation and deposition of ECM proteins in the lungs. Hence, Boveda-Ruiz et al. [12] concluded that Tregs exert different roles in the early and late stages of pulmonary fibrosis. Taken together, these data suggest that the application of Tregs with animals in fibrotic stages is more likely to reflect clinical settings where therapy is initiated against established pulmonary fibrosis.

IL-10 has been shown to reduce many inflammatory reactions [36] and is thus recognized as an anti-inflammatory cytokine possessing immunosuppressive properties. Arai et al. demonstrated that introduction of the IL-10 gene into mice inhibited BLM-induced lung injury in vivo [25], and a similar effect of IL-10 was confirmed by Nakagome et al. [26]. Conversely, IL-10 overexpression has been linked to the development of pulmonary fibrosis in vivo [37]. Thus, the exact contribution of IL-10 to the development of pulmonary fibrosis in vivo remains undetermined. With respect to IL-10 production in the lungs, the results of several in vivo studies have shown that IL-10 levels were unchanged or decreased by BLM administration [38, 39] while increased IL-10 protein concentrations were found in the plasma of treated animals [40]. We also observed a significant increase in plasma IL-10 levels in mice after a BLM challenge. Although we expected a further increase in IL-10 production by Treg transfer, plasma IL-10 levels were not changed, as compared with those in a saline-treated group. These data imply that increased IL-10 levels in mice receiving BLM may serve as a protective mechanism that may be overcome by a severe injury that ultimately results in fibrosis. To further explore the potential of IL-10 in the current model, we adoptively transferred Tregs from IL-10 knock-out mice. The results suggested that IL-10 from Tregs might have an impact on the resolution of BLM-induced pulmonary fibrosis, which supports the data from previous studies [25, 26]. Taken together, IL-10 could be considered to work not only as an anti-inflammatory but also as an anti-fibrotic mediator.

In this study, we removed spleens at different stages in a BLM-induced murine pulmonary fibrosis model. Most Foxp3^+^ natural Treg cells are produced by the thymus as an antigen-primed and functionally mature T cell subpopulation specialized for immune suppression [41]. Subsequently, they distribute to the peripheral lymph nodes and spleen. Monocytes have been reported to be produced in the bone marrow from hematopoietic precursors at steady state; however, during inflammation, the spleen is also involved in the generation, depot, and deployment of these cells [42]. Although BLM administration can reduce the splenic lymphocyte pool size [30], it is not known whether the spleen is involved in Treg deployment in this model. However, our current observation demonstrated that spleen removal at an early stage of the current model changed the course of BLM-induced murine pulmonary fibrosis. Moreover, the results were similar in part to those reported by Boveda-Ruiz et al., where removal of Tregs during the inflammatory phase ameliorates fibrosis [12]. These findings raised the possibility that the spleen is a reservoir of Tregs and contributes to disease progression.

This study has at least 3 principal limitations. First, although BLM-induced lung fibrosis is the best characterized and a widely used animal model for evaluating the therapeutic efficacy of experimental agents [34], it is impossible to completely recapitulate human IPF. However, experiments with BLM-induced pulmonary fibrosis in vivo have been utilized for the development of currently widely used anti-fibrotic agents, including pirfenidone [43] and nintedanib [44]. Hence, investigating the therapeutic efficacy in vivo with BLM-induced pulmonary fibrosis model appears to be an appropriate surrogate strategy for developing novel therapeutics. Second, the discrepant observations reported as the results of Treg administration may be attributed to different routes of BLM administration. Because various means of BLM challenge are available (including the intratracheal, intravenous, and intraperitoneal routes, as well as an osmotic pump as employed in this study), it is likely that differences in protocols used to induce lung fibrosis by BLM could explain the discrepant observations among the studies. Lastly, we could not clarify the detailed mechanisms by which Tregs resolve fibrosis in the current study. The role of Tregs was originally considered to suppress immune responses, although we could not directly demonstrate this point in the current model. However, recent reports suggest that Tregs have roles in tissue repair through the production of growth factors including keratinocyte growth factor (KGF) or amphiregulin [45], which have the potential to restore alveolar epithelial cell damage [46]. Therefore, studies to elucidate the tissue repair mechanisms promoted by Tregs should be considered using the current fibrosis model. However, such studies should be performed carefully because a recent report demonstrated that KGF treatment in patients with acute respiratory distress syndrome caused rather harmful effects [47].

## Conclusions

In conclusion, Tregs significantly resolved BLM-induced murine pulmonary fibrosis when adoptively transferred in the fibrotic phase of the current model, which implicates the anti-fibrotic properties of Tregs in the chronic stage of the disease. Although the current study involved a single injection of Tregs during the course of the disease, repeated administration or ex vivo expansion of these cells is warranted to investigate the development of cell-based therapy for IPF.

## Abbreviations

BLM: Bleomycin
BSA: Bovine serum albumin
CCL2: Chemokine (CC motif) ligand-2
DAPI: 4′,6-diamidino-2-phenylindole
ECM: Extracellular matrix
ELISA: Enzyme-linked immunosorbent assay
FGF: Fibroblast growth factor
HE: Hematoxylin and eosin
IL: Interleukin
IPF: Idiopathic pulmonary fibrosis
KGF: Keratinocyte growth factor
mAb: Monoclonal antibody
PBS: Phosphate-buffered saline
Tregs: Regulatory T cells
